# Resveratrol and its analogs suppress HIV replication, oxidative stress, and inflammation in macrophages

**DOI:** 10.1515/nipt-2023-0012

**Published:** 2023-07-13

**Authors:** Santosh Kumar, Namita Sinha, Sunitha Kodidela, Sandip Godse, Bhupesh Singla, Udai P. Singh, Hari K. Bhat

**Affiliations:** Department of Pharmaceutical Sciences, College of Pharmacy, The University of Tennessee Health Science Center, Memphis, TN, USA; Department of Pharmacology and Pharmaceutical Sciences, School of Pharmacy, University of Missouri-Kansas-City, Kansas City, MO, USA

**Keywords:** HIV, inflammation, macrophages, oxidative stress, resveratrol

## Abstract

**Objectives:**

HIV suppression in brain viral reservoirs, especially macrophages, and microglia is critical to suppress HIV neuropathogenesis and subsequently HIV-associated neurocognitive disorders (HAND). Since most antiretroviral therapy (ART) drugs do not achieve optimal therapeutic concentrations in the brain and can cause neurotoxicity, an alternative/adjuvant therapy is needed to suppress HIV neuropathogenesis. In this study, our objectives were to examine the anti-HIV, antioxidant, and anti-inflammatory potential of resveratrol (RES) and its synthetic analogs 4-(E)-{(p-tolylimino)-methylbenzene-1,2-diol} (TIMBD) and 4-(E)-{(4-hydroxyphenylimino)-methylbenzene,1,2-diol} (HPIMBD) in HIV-infected macrophages.

**Methods:**

We used HIV replication (viral load), oxidative stress (reactive oxygen species and antioxidant enzymes), and inflammatory response (pro- and anti-inflammatory cytokines/chemokines) assays to achieve the objectives of the study.

**Results:**

Our results showed that RES and its analogs HPIMBD and TIMBD at 25 µM concentration significantly decrease HIV replication in both primary monocyte-derived macrophages and U1-differentiated macrophages. Moreover, RES and its analogs do not induce any cytotoxicity for up to 3 days in these cells. Further, treatment with RES and TIMBD (25 µM) also reduced the levels of reactive oxygen species without affecting the expression of antioxidant enzymes, SOD1, and catalase in U1 macrophages. Besides, RES and HPIMBD treatment inhibited the proinflammatory cytokines and chemokines in U1 macrophages, which was associated with decreased levels of anti-inflammatory cytokines. Importantly, our western blot experiments show that RES also decreases cellular proinflammatory cytokine IL-1β, which is usually elevated in both myeloid and neuronal cells upon HIV infection.

**Conclusions:**

Taken together, our results suggest that RES and/or its analogs are important adjuvants that may be used not only to suppress HIV but also oxidative stress and inflammation in brain viral reservoirs.

## Introduction

HIV-infected macrophages infiltrate the brain and spread the virus into perivascular macrophages and microglia, which subsequently cause HIV-associated neuropathogenesis including HIV-associated neurocognitive disorders (HAND) [1]. HAND significantly impacts the quality of life, especially in older populations [2, 3]. In the era of antiretroviral therapy (ART), although the prevalence of HIV-associated dementia has significantly reduced, asymptomatic and mild HAND persist in approximately >50 % of the HIV-infected population [4]. Further, despite the success of ART in reducing morbidity and mortality, its effective treatment outcomes only occur in 1/3rd of the HIV population.

Reaching adequate ART drug concentrations in the brain is essential for treating neuroHIV and HAND. However, due to a highly selective blood-brain barrier (BBB), many ART drugs have restricted entry into the brain [5]. Furthermore, efflux transporters in the BBB, macrophages, and microglia [6–8] cause suboptimal concentrations of ART drugs in the brain. The suboptimal concentrations of these drugs in the brain can also be due to the presence of a metabolic enzyme, cytochrome P450 3A4 (CYP3A4) in brain cells [9, 10]. As a result, there is limited suppression of viral replication in brain macrophages and microglia, which contributes to a critical barrier in treating HIV neuropathogenesis including HAND. Further, there is a need to carefully monitor the drug-associated toxicities as some ART drugs are neurotoxic and can exacerbate HIV-associated CNS pathologies [11]. Therefore, it is important to find natural compounds or nutraceuticals, which can serve as adjuvants to boost antiviral function and have an anti-HIV activity.

Plant-derived phytoestrogens have been widely studied for their therapeutic effects under different pathological conditions [12]. Resveratrol (RES), one of the most studied phytoestrogens, has a therapeutic activity in various neuronal disorders, especially Alzheimer’s disease [13]. Studies have shown that RES confers its therapeutic potential by suppressing oxidative stress, in part, by modulating antioxidant defense mechanisms [14, 15]. Furthermore, some clinical reports have proposed that RES may be a potential candidate for the prevention and/or treatment of HIV/AIDS, which reduces viral replication by 20–30 % [16]. Another study has demonstrated that RES completely blocks HIV infection at a low micromolar dose in resting CD4^+^ T cells [17], demonstrating it as a potential adjuvant in anti-HIV therapy, especially in CNS reservoirs [18]. Although RES has shown therapeutic effects including in HIV pathogenesis, its poor specificity and bioavailability have presented a challenge for its clinical use [19]. Therefore, RES derivatives have been synthesized and examined for their ability to enhance the antiviral activity of decitabine, a nucleoside analog [20]. However, of the six RES derivatives tested in this study, none had anti-HIV activity greater than RES.

Our group has attempted to assess the anti-HIV activity of a different group of novel RES-analogs. We have synthesized and developed novel analogs of RES, 4-(E)-{(p-tolylimino)-methylbenzene-1,2-diol} (TIMBD) and 4-(E)-{(4-hydroxyphenylimino)-methylbenzene,1,2-diol} (HPIMBD) [21]. Our previous studies have shown that both HPIMBD and TIMBD have potent antioxidant effects using breast cancer cell lines [22]. Further, our previously reported study also suggests that TIMBD has the potential to decrease HIV-gp120-induced expression of inflammatory cytokines in astrocytes [23]. Therefore, in this study, we have examined the effects of RES, HPIMBD, and TIMBD in suppressing HIV pathogenesis, including oxidative stress and inflammation in HIV-infected macrophages.

## Materials and methods

### Chemicals and reagents

RES was bought from Sigma-Aldrich (St. Louis, MO). RES analogs TIMBD and HPIMBD were synthesized and purified as reported previously [21]. RES and its analogs were dissolved in dimethyl sulfoxide (DMSO) before treatments. The final concentration of DMSO in all experiments was 0.1 %. The HIV Type 1 p24 Antigen ELISA kit to measure the viral load was purchased from ZeptoMetrix Corporation (Buffalo, NY). Pierce Lactate Dehydrogenase (LDH) Cytotoxicity Assay Kit and fluorescent dye 5-(and-6)-chloromethyl 2′,7′- dichlorodihydrofluorescein diacetate (CM-H2DCFDA) were purchased from ThermoFisher Scientific (Grand Island, NY). Cell culture reagents including the Roswell Park Memorial Institute (RPMI) 1640 media were bought from Corning Inc. (Tewksbury, MA). Heat-inactivated Fetal bovine serum (FBS) was procured from Atlanta Biologicals (Atlanta, GA). L-glutamine was purchased from Fisher Scientific.

### Cell culture and treatment

U1 cells, which are chronically HIV-1-infected U937 cells, were procured from the NIH AIDS Reagent Program (Germantown, MD). The U1 cell lines have been extensively used by our group and other researchers to study the role of drug abuse and nutraceuticals in HIV replication [24–30]. Further, the results obtained using these cells have been largely replicated in HIV-infected primary macrophages [24, 25, 30]. The U1 cells were cultured in RPMI 1640 media supplemented with 10 % FBS and 1 % L-glutamine. The U1 cells were differentiated into macrophages (0.3 million cells in 0.4 mL) by treatment with 100 nM phorbol 12-myristate 13-acetate for 3 days. After 3 days of differentiation, the media was removed, and cells were washed with phosphate buffer saline (PBS) before adding fresh RPMI media to the differentiated cells. The cells were then incubated for 3–4 h before starting any treatments. Differentiated cells were treated with control (DMSO) and RES/their analogs (TIMBD and HPIMBD) at 10 µM for 2 days followed by an optimized concentration of 25 μM for 2–3 days.

Human monocyte-derived macrophages (MDM) were prepared as described in our previous study [24]. Briefly, peripheral blood mononuclear cells (PBMCs) were collected from buffy coats (Interstate blood bank, Memphis, TN) following density-gradient separation using the established protocol [31, 32]. PBMCs were allowed to adhere to the plastic for 4 h, which were then removed and cultured in media supplemented with M-CSF (25 µM) for 7–10 days to facilitate their differentiation into macrophages. MDM was then collected and treated with polybrene for 30 min followed by infection with HIV-Ada strain (20 ng/10^6^). Cells were seeded in 6-well plates and fresh media was added every third day to ensure cell viability. The p24 antigen levels were determined in the collected cell culture supernatant using the p24 ELISA kit as described below. Upon confirming viral infection (7–10 days), HIV-infected MDM was treated with control (DMSO) or RES/its analogs (25 μM).

### HIV p24 ELISA

HIV p24 antigen levels in the supernatant were measured using the HIV-1 p24 Antigen ELISA kit (Zeptometrix Corporation, Buffalo, NY) according to the manufacturer’s recommendation. Briefly, 25 µL of the media sample was added to the wells containing the p24 viral antigen. It was then sequentially incubated with biotin-labeled human antibody to HIV for 1 h, streptavidin-conjugated horseradish peroxidase for 30 min at 37 °C, and tetramethylbenzidine substrate for 30 min in the dark. The absorbance of every well was measured at 450 nm and compared against the standard curve to determine the p24 (pg/mL) levels in the samples. The viral load was expressed as a percentage of HIV p24 levels observed in DMSO-treated control wells.

### LDH cytotoxicity assay

Cytotoxicity was measured in cell culture supernatant collected from macrophages using the Pierce Lactate Dehydrogenase (LDH) Cytotoxicity Assay Kit following the manufacturer’s protocol. Briefly, 50 µL cell culture supernatant was mixed with 50 μL LDH reaction mixture in a 96-well plate, and the plate was incubated at room temperature for 30 min. Then, 50 µL LDH stop solution was added before measuring the fluorescence at *λ*_ex_ of 490 nm and *λ*_em_ of 680 nm using a microplate reader (Cytation™ 5 Cell Imaging Multi-Mode Reader, BioTek, VT). A higher absorbance value suggests higher cytotoxicity.

### Measurement of reactive oxygen species (ROS)

ROS was quantified by flow cytometry analysis using a fluorescence probe CM-H_2_DCFDA, which reacts with various ROS. Cells after indicated treatment times were washed twice with PBS, and CM-H_2_DCFDA solution (5 µM) in PBS was added. Cells were then incubated at room temperature in the dark for 45 min, washed, and resuspended in 300 μL PBS. Intracellular ROS generation was detected using flow cytometry analysis (Agilent NovoCyte Cytometer).

### Western blotting

To determine the expression levels of cytokines, chemokines, and antioxidant enzymes (AOEs), an equal amount of protein (10 µg) was separated using SDS-polyacrylamide gel (10 % gel), run for 90 min at 150 V, and then transferred to polyvinyldene fluoride membranes using a current of 0.35 Amp for 90 min. Membranes with transferred proteins were blocked with 10 mL of Li-Cor blocking buffer (LI-COR Biosciences, Lincoln, NE) for 1 h to avoid any nonspecific binding of antibodies. Then, the membrane was incubated overnight at 4 °C with target primary antibodies (IL-1β Rabbit Pab, 1:500 dilution, Proteintech, catalog#16806-1-AP; TNF-ά Rabbit Pab, 1:500 dilution, Abcam, catalog # ab6671; MCP-1 Mouse Mab, 1:400 dilution, Proteintech, catalog# 66271-1-1g; IL-6 Rabbit Pab, 1:500 dilution, Proteintech, catalog# 21865-1-AP; IL-1RA Rabbit Pab, 1:500 dilution, Proteintech, catalog# 10844-1-AP; SOD1 Mouse Mab, 1:700 dilution, Santa Cruz Biotechnology, catalog #sc-101523; Catalase mouse Mab, 1:700 dilution, Santa Cruz Biotechnology, catalog # sc-365738; β-Actin Mouse mAb.1:4000 dilution, Cell Signaling, Catalog #3700). After overnight incubation, the blots were washed and incubated with the appropriate secondary antibodies (Goat anti-Mouse Mab, Goat anti-Rabbit Mab, 1:10000 dilution, LI-COR Biosciences) and membranes were scanned using an Odyssey DLx Infrared Imaging System. The densitometric data was obtained from the Image Studio Lite software. Actin was used as an internal loading control to normalize the expression of these proteins.

### Cytokine analysis

The protein levels of the cytokines (TNF-α, IL-1β, IL-8, IL-6, IL-1RA, IL-10) and chemokines (MCP-1 and RANTES) were measured in the cell culture media (25 µL) of U1 macrophages using Human Custom Procartaplex 8-plex (Invitrogen, ThermoFisher Scientific, NY). According to the manufacturer’s instructions, samples, standards, and magnetic beads were added to the 96-well ELISA plate and mixed on a plate shaker for 1 h at room temperature. The plate was then incubated at 4 °C overnight. The beads were washed followed by the addition of detection antibody, and measurement of expression levels of cytokines/chemokines was performed (pg/mL) using a Magpix system (Austin, TX). The data were analyzed using an xPONENT^®^ software.

### Statistical analysis

GraphPad Prism 9 (GraphPad Software; La Jolla, CA) was used to perform all statistical analyses and plot graphs. The data are presented as Mean ± SEM. One-way ANOVA with Tukey’s post-hoc test was applied to compare multiple groups. A p≤0.05 was considered statistically significant.

## Results

### RES analogs HPIMBD and TIMBD reduced HIV replication in primary macrophages

**Figure 1:**
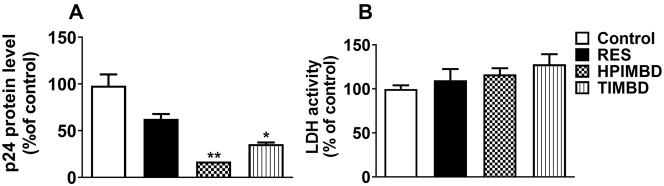
Effect of RES and its analogs HPIMBD and TIMBD on HIV replication (A) and cytotoxicity (B): monocyte-derived macrophages were treated with DMSO control, RES (25 μM), HPIMBD (25 μM), and TIMBD (25 μM). HIV p24 protein expression levels were measured at the end of the treatment using an ELISA kit. Cytotoxicity was measured using an LDH assay kit. One-way ANOVA with Tukey’s post-hoc test was applied to compare between multiple groups. *, and ** represent p≤0.05 and p≤0.01, respectively, when compared to control. Results are expressed as mean ± SEM of n=6.

First, we determined whether RES and its two analogs HPIMBD and TIMBD reduce HIV replication in primary MDM. We treated MDM with RES, HPIMBD, and TIMBD for two days at 25 µM concentration, which was established by performing dose-response studies (10 and 25 µM) in U1 macrophages. Our results suggest that RES, though not statistically significant, and its analogs HPIMBD (p≤0.01) and TIMBD (≤0.05) significantly reduced protein expression levels of p24 compared to the control treatment (Figure 1A). Importantly, the reduction in the viral load was higher with treatment with both RES analogs than with the parent compound RES (Figure 1A). Further, we also determined whether these compounds exert any cytotoxicity. The cytotoxicity data obtained using LDH assay showed no significant cytotoxicity for up to 2 days (Figure 1B). Taken together, these results from the primary macrophages established that RES and its analogs HPIMBD and TIMBD inhibit HIV replication without inducing cytotoxicity in macrophages.

### RES and its analogs HPIMBD and TIMBD suppressed HIV replication in U1 macrophages

**Figure 2:**
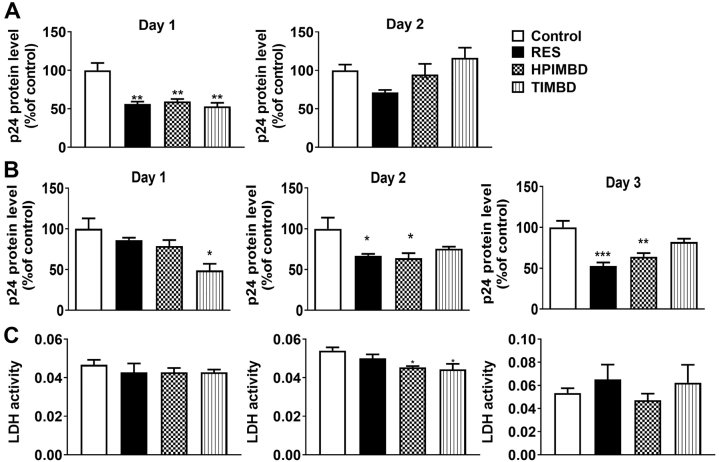
Effect of RES and its analogs HPIMBD and TIMBD on HIV replication at 10 µM (A) and 25 µM (B), and cytotoxicity at 25 µM (C) in U1 differentiated macrophages: the U1 macrophages were treated with DMSO (control), RES (10, 25 μM), HPIMBD (10, 25 μM), and TIMBD (10, 25 μM) for three days. HIV p24 protein expression levels were measured each day using an ELISA kit. Cytotoxicity was measured using an LDH assay kit. One-way ANOVA with Tukey’s post-hoc test was applied to compare between multiple groups. *, **, and *** represent p≤0.05, p≤0.01, and p≤0.001, respectively, when compared to control. Results are expressed as mean ± SEM of n=6.

To further examine the role of RES and its analogs on HIV pathogenesis, which includes HIV replication, cytotoxicity, oxidative stress, and cellular and systemic inflammation, we used an established cell line model (U1 macrophages) for primary macrophages. First, we investigated the effects of RES, HPIMBD, and TIMBD at 10 µM on HIV replication in U1 macrophages for 2 days. Our results showed that 10 µM of RES, HPIMBD and TIMBD significantly reduced HIV replication (p≤0.01) after 1 day of treatment, but not after 2 days of treatment (Figure 2A). We then treated U1 macrophages with RES, HPIMBD, and TIMBD at 25 μM for 3 days and measured the p24 levels. We observed that RES and its analog, HPIMBD, significantly reduced p24 levels on day 2 (p≤0.01) and day 3 (p≤0.0001, 0.001 respectively) (Figure 2B), but not on day 1. Whereas another analog of RES, TIMBD, significantly reduced p24 levels on day 1 but did not suppress HIV replication after 2 and 3 days of treatment (Figure 2B). Although RES and its analogs showed varying levels of HIV suppression in primary MDM and U1 cell line macrophages, RES and HPIMBD appeared to show more consistent HIV suppression. Further, like primary macrophages, we determined whether these compounds exert any cytotoxicity for 3 days in HIV-infected macrophages. The data obtained with cytotoxicity experiments showed no significant toxicity by any of these compounds, except for some marginal decrease on day 2 by HPIMBD and TIMBD (Figure 2C). The results suggest that RES and its analogs HPIMD and TIMBD are safe at 25 µM for up to 3 days. Since RES, HPIMD, and/or TIMBD at 25 µM showed reduced HIV replication with no cytotoxicity for up to 3 days, we chose this concentration in the subsequent experiments.

### RES and TIMBD reduced oxidative stress in U1 macrophages

**Figure 3:**
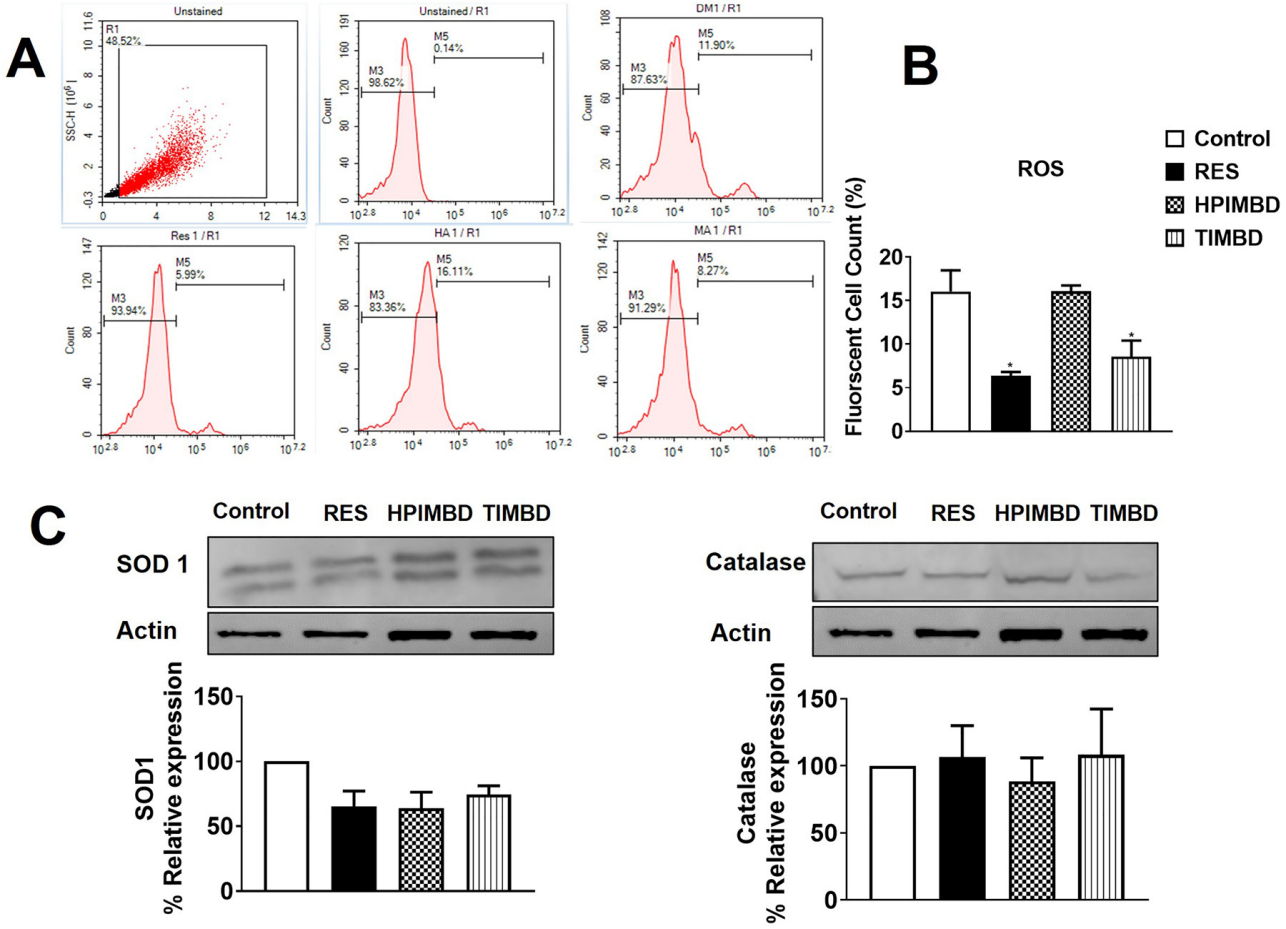
Effect of RES and its analogs HPIMBD and TIMBD on ROS (A–B) and antioxidant enzymes (catalase and SOD1) (C) in U1 differentiated macrophages. (A–B) ROS measurements: U1 macrophages were treated with DMSO, RES (25 μM), HPIMBD (25 μM), and TIMBD (25 μM) for 2 days, and cells were harvested at the end of the treatment. Treated cells were stained with CM-DCFDA dye and the fluorescence was measured using flow cytometry at excitation/emission of 495/519 nm. U1 macrophages were treated with DMSO, RES (25 μM), HPIMBD (25 μM), and TIMBD (25 μM) for 3 days and cells were harvested at the end of the treatment. The expression levels of catalase and SOD1 proteins were measured by western blot. One-way ANOVA with Tukey’s post-hoc test was applied to compare between multiple groups. Results are expressed as means ± SEM of n=3–4 experiments. * Represent p<0.05 when compared to control.

Since oxidative stress is a hallmark of HIV pathogenesis, especially in neuronal damage, we determined whether RES and its analogs HPIMBD and TIMBD alter ROS production. We treated U1 macrophages with RES, HPIMBD, and TIMBD (25 μM) for two days and measured ROS generation. We observed that RES and its analog, TIMBD, but not HPIMBD, significantly reduced ROS levels (p<0.01) (Figure 3A and B). We then measured the levels of oxidative stress marker proteins (AOEs)such as catalase and SOD1, to determine whether their levels are altered upon treatments with RES and its analogs. Although there was a trend in a decrease in the levels of SOD1 upon treatments with RES, HPIMBD, and TIMBD, neither SOD1 nor catalase showed a significant decrease by the treatments (Figure 3C). Overall, the results suggest that RES and TIMBD reduce oxidative stress via attenuating ROS production without affecting the levels of AOEs that we investigated.

### RES and its analogs HPIMBD and TIMBD differentially reduced systemic inflammation in U1 macrophages

**Figure 4:**
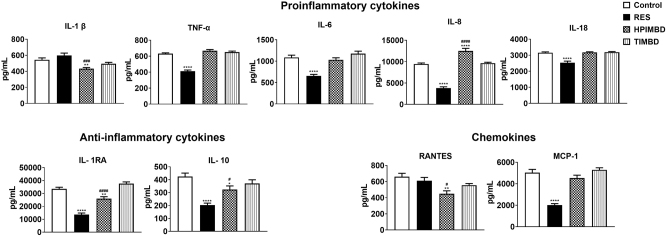
Effect of RES and its analogs on pro-inflammatory cytokine (IL-1β, TNFα, IL-6, IL-8, IL1-8), anti-inflammatory cytokine (IL-10, IL-1RA), and chemokines (RANTES, MCP-1) in U1 macrophages. U1 macrophages were treated continuously with DMSO (control), RES (25 μM), HPIMBD (25 μM), and TIMBD (25 μM) for three days. Cytokines and chemokines were measured using multiplex ELISA methods. One-way ANOVA with Tukey’s post-hoc test was applied to compare between multiple groups. *, **, ***, and **** represent as p≤0.05, p≤0.01, p≤0.001, and p≤0.001, respectively, when compared to control. ##, and ### represent p≤0.01, and p≤0.001, respectively when compared to RES. Results are expressed as mean ± SEM of n=6.

HIV-infected myeloid cell-mediated neuroinflammation is another hallmark of HIV neuropathogenesis, which subsequently causes neuronal damage and HAND. Therefore, we determined whether RES and its analogs HPIMBD and TIMBD alter the secretion of proinflammatory cytokines (IL-1β, TNF-α, IL-6, IL-8, and IL-18), chemokines (MCP-1 and RANTES), and the secretion of anti-inflammatory cytokines (IL-1RA and IL-10) in U1 macrophages. We observed was that treatment with RES resulted in the alteration of most cytokines and chemokines (Figure 4). Specifically, RES treatment caused a significant decrease in the levels of pro-inflammatory cytokines TNF-α, IL-6, IL-8, and IL-18, and chemokines MCP-1. However, RES also decreased the level of anti-inflammatory cytokines IL-1RA and IL-10 (Figure 4). Interestingly, HPIMBD treatment significantly decreased the pro-inflammatory cytokines IL-1β but resulted in an increase in the levels of IL-8. Moreover, it also decreased the levels of chemokine RANTES (Figure 4). Like RES, HPIMBD treatment also resulted in a decrease in the levels of anti-inflammatory cytokines IL-1RA and IL-10. However, TIMBD did not alter the levels of any cytokines and chemokines. Overall, the results suggest that RES and HPIMBD significantly decrease pro-inflammatory cytokines and chemokines, and overall reduced inflammation, which is consistent with reduced HIV replication (Figure 2 vs. Figure 4).

### RES and its analogs HPIMBD and TIMBD reduced cellular inflammation in U1 macrophages

**Figure 5:**
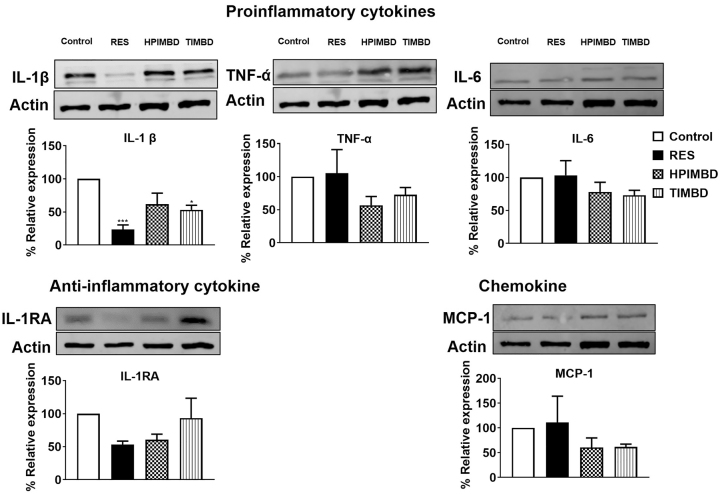
Effect of RES and its analogs HPIMBD and TIMBD on cytokines/chemokines in U1 macrophages. U1 macrophages were treated with DMSO, RES (25 μM), HPIMBD (25 μM), and TIMBD (25 μM) for 3 days and cells were harvested at the end of the treatment. The expression of pro-inflammatory: IL-1β, TNF-α, IL-6; anti-inflammatory: IL-1ra, and chemokine: MCP-1 proteins were measured by western blot. One-way ANOVA with Tukey’s post-hoc test was applied to compare between multiple groups. Results are expressed as means ± S.E.M of n=4 experiments. * and *** represents p<0.05 and p<0.001 when compared to control.

Next, we determined whether RES and its analogs can decrease intracellular pro- and anti-inflammatory cytokines and chemokines protein levels in U1 macrophages. For this, we used selected proinflammatory cytokines (IL-1β, TNF-α, and IL-6), anti-inflammatory cytokine (IL-1RA), and chemokine (MCP-1). The major findings from these experiments were that RES and TIMBD, but not HPIMBD, significantly reduced the protein expression of IL-1β (Figure 5) in U1 macrophages. Although HPIMBD and TIMBD showed a trend towards reducing the levels of proinflammatory cytokines TNF-α and IL-6 and chemokine MCP-1, the deceases were not statistically significant (Figure 5). Overall, these results suggest RES and its analogs do cause decreased cellular inflammation in macrophages.

## Discussion

Most ART drugs not only fail to access the brain reservoirs, especially brain macrophages and microglia but are neurotoxic at their therapeutic doses. Therefore, it is highly desirable to find an alternative/adjuvant therapy that not only has anti-HIV but also antioxidant and anti-inflammatory characteristics and can enter the brain. This is the first report of its kind that shows the effect of RES and its analogs HPIMBD and TIMBD on anti-HIV, antioxidant, and anti-inflammatory activities in macrophages. The findings have clinical relevance because RES or its analogs alone or by encapsulating in a nanocarrier could potentially be used as adjuvant therapy with ART drugs to suppress HIV neuropathogenesis.

Several nutraceuticals including RES have been the subject of study as adjuvant therapy for HIV treatment in the past [16]. To overcome the relatively low solubility and bioavailability of RES, several derivatives of RES have been synthesized, however, they did not improve HIV pathogenesis [19]. Furthermore, attempts were made to encapsulate RES in synthetic nanoparticles for its targeted delivery at the therapeutic level [16]. However, due to the immunogenicity of the synthetic nanoparticles and no improvement in anti-HIV activity, the nanocarrier-based approach for RES was halted. Recently, the synthesis of two derivatives of RES, namely HPIMBD, and TIMBD, which have higher bioavailability, antioxidant, anti-inflammatory, and anti-cancer activities than RES, reignited the interest to examine their role in anti-HIV activity [21, 22]. Furthermore, the recent development of novel natural nanoparticles, EVs, that can carry ART drugs and nutraceuticals to the brain and target viral reservoirs rejuvenated the use of nutraceuticals to potentially suppress HIV neuropathogenesis [33].

Our group has recently shown the importance of nutraceuticals in suppressing HIV in brain viral reservoirs [33]. We have recently shown the role of cucurbitacin-D in suppressing HIV replication in macrophages [26]. The anti-HIV activity of cucurbitacin-D is comparable to that obtained by darunavir/ritonavir, which is one of the drug regimens that are used in common HIV treatment. Cucurbitacin-D not only suppresses HIV replication in macrophages but also decreases inflammation. In this context, significant suppression of HIV replication in both primary MDM and U1 macrophages by RES and its analogs, especially HPIMBD, is an important finding. Furthermore, no change in cytotoxicity by RES or its analogs further suggests that RES and its analogs would be relatively safe for HIV treatment. Our findings are consistent with the clinical reports that RES may be a potential candidate for the prevention and/or treatment of HIV/AIDS [16]. Furthermore, our results are also consistent with the recent finding that RES completely blocks HIV infection in resting CD4 T cells [17].

The increase in oxidative stress (ROS) and modulation of AOEs in HIV-infected myeloid cells and their subsequent release that are taken up by neuronal cells is one of the major characteristics of HIV neuropathogenesis [34]. In addition, oxidative stress in myeloid cells plays a major role in microglial activation and cell survival [35]. The oxidative stress agents generated by HIV-infected myeloid cells can be taken up by neuronal cells directly or by packaging them in extracellular vesicles (EVs) [36]. Our previous studies have shown the role of EVs in packaging AOEs and delivering them to other cells including neuronal cells via intra- and intercellular communication [28, 37, 38]. More specifically, one study has shown that EVs containing AOEs, released from uninfected or HIV-infected macrophages, when exposed to HIV-infected macrophages can alter HIV pathogenesis [38]. In this context, RES and TIMBD that show >50 % reduction in ROS in macrophages may also reduce oxidative stress in neuronal cells. The findings have clinical relevance because ART drugs cause increased oxidative stress and the use of RES or TIMBD as an adjuvant can help reduce oxidative stress in macrophages and perhaps in neuronal cells as well. The findings are consistent with the literature that RES has a protective role against neurodegenerative disorders by reducing oxidative stress [39]. Thus, RES has been a focus of study as a neuroprotective agent in several neurodegenerative diseases [40]. Furthermore, a meta-analysis study has shown that RES has positive effects on diabetic nephropathy by inducing the activities of AOEs, such as SOD, CAT, GSH, and GPx [41]. With regards to RES analogs, HPIMBD and TIMBD, the current findings are consistent with our earlier study that both analogs have potent antioxidant effects in breast cancer cells [22].

Inflammatory cytokines and chemokines play a major role in modulating inflammation in myeloid cells, especially in context to HIV neuropathogenesis [42]. In addition, their release in biofluids can also cause inflammation in neuronal cells via systemic circulation [43, 44]. While proinflammatory cytokines (IL-1β, TNF-α, IL-6, IL-8, and IL-18) and chemokines (MCP-1 and RANTES) can increase inflammation, anti-inflammatory cytokines (IL-1RA and IL-10) can decrease inflammation. Like viral proteins, these inflammatory agents can also be taken up by neurons directly or by packaging in EVs and modulate neuronal inflammation [45]. Our previous studies have shown the role of EVs in packaging inflammatory agents, especially IL-1β, followed by their delivery to recipient cells including neuronal cells [28, 37]. In this context, RES and HPIMBD which show a reduction in many pro-inflammatory cytokines and chemokines can reduce inflammation in macrophages. Furthermore, these cytokines and chemokines, released from macrophages, can be packaged in EVs, and delivered to neurons reducing neuroinflammation. On the other hand, a reduction in anti-inflammatory cytokines by RES and HPIMBD may be incapable of protecting the cells from inflammation. The overall inflammation depends on the relative changes in pro-inflammatory cytokines/chemokines and anti-inflammatory cytokines. In this study, RES and HPIMBD are expected to decrease overall systemic inflammation, and perhaps neuroinflammation, because many proinflammatory cytokines and chemokines are decreased in U1 macrophages after RES and HPIMBD treatment. However, only IL-1RA anti-inflammatory cytokine is decreased in macrophages. Although IL-10 is generally an anti-inflammatory cytokine, it can also be pro-inflammatory under certain conditions in different cell types [46].

Our recent reports demonstrated the role of IL-1β in HIV-infected macrophages, astrocytes, and neurons via EV-mediated intercellular interactions [37, 47]. In these studies, we have shown that IL-1β is significantly increased upon HIV infection in macrophages in all these cells. We have also shown that EV-containing IL-1β, isolated from HIV-infected macrophages, when exposed to astrocytes and neuronal cells further increases the levels of cellular IL-1β in astrocytes and neurons. In this context, the current study has shown that both cellular and systemic IL-1β are significantly reduced by RES. These findings have a major impact on the benefits of RES in suppressing inflammation, not only in HIV-infected macrophages but also in astrocytes and neurons. Reduced inflammation in both HIV-infected macrophages and astrocytic and neuronal cells can reduce neuroinflammation and subsequently reduce neuronal damage and HAND. The findings are consistent with the kinds of literature that RES has neuroprotective effects via decreasing the levels of IL-1β in many disease conditions including neuronal and cognitive diseases [13, 48]. A meta-analysis study has shown that RES has positive effects in diabetic nephropathy by reducing the levels of IL-1β, but no effect on IL-6 and TNF-α levels [41].

The role of RES as an immunoregulator in tissue protection and tumor suppression is well known [49]. The literature suggests that RES can regulate various cellular signaling events including cytokines/chemokines secretion and the expression of several other immune-related genes in the tumor microenvironment [50]. Thus, there have been consistent efforts in developing RES as a nutraceutical for many diseases [51]. A recent report has also shown that RES regulates neuroinflammation and induces adaptive immunity in Alzheimer’s disease [52] and therefore has been suggested to have therapeutic activity for Alzheimer’s disease [13, 53]. With regards to RES analog, TIMBD, the current findings are consistent with our earlier study that TIMBD decreases HIV-gp120-mediated-inflammatory cytokines in astrocytes [23].

Overall, we show that RES and its analogs demonstrate significant anti-HIV activity, antioxidant properties, and reduce both cellular and systemic inflammation in macrophages. A comparative assessment of RES analogs for HIV replication and inflammatory response indicates that HPIMBD is most consistent on suppressing HIV replication and reducing inflammatory cytokines (IL-1β and IL-18) and chemokine (RANTES). We do acknowledge that TMIBD also showed HIV suppression in day 1 treatment. Since RES has less solubility and bioavailability, the relatively high solubility and bioavailability of its analogs, especially HPIMBD, can have an impact as an adjuvant in HIV therapy. We are yet to perform pharmacokinetic (PK) studies of HPIMBD and TMIBD. However, based on RES PK study [54], we expect that 15 mg/kg intravenous or 50 mg/kg oral dose in mice would be non-toxic and yield 5–10 µM HPIMBD in plasma circulation. Our study shows that 10 µM HPIMBD is sufficient to suppress HIV replication in macrophages. If HPIMBD does not reach its therapeutic concentration in plasma, it can be nano formulated for drug delivery and targeted to myeloid cells, especially brain macrophages and microglia.

This is a very important finding because persistent HIV replication in myeloid cells including brain macrophages subsequently release viral proteins, oxidative stress, and inflammatory agents and cause neuronal damage. There are no ART drugs that can sufficiently permeate brain and suppress HIV in brain macrophages and microglia. Findings that HPIMBD can suppress HIV and reduce inflammatory response in macrophages is a step forward in its use as adjuvant therapy either directly as oral dose or using targeted nanoformulation. However, before realizing its potential as adjuvant therapy, a complete PK, pharmacodynamics, and safety profile studies need to be performed in animal models.
